# Steeper Iris Conicity Is Related to a Shallower Anterior Chamber: The Gutenberg Health Study

**DOI:** 10.1155/2017/2190347

**Published:** 2017-09-11

**Authors:** Alexander K. Schuster, Norbert Pfeiffer, Stefan Nickels, Andreas Schulz, Philipp S. Wild, Maria Blettner, Karl Lackner, Manfred E. Beutel, Thomas Münzel, Urs Vossmerbaeumer

**Affiliations:** ^1^Department of Ophthalmology, University Medical Center of the Johannes Gutenberg-University Mainz, Mainz, Germany; ^2^Preventive Cardiology and Preventive Medicine, Center for Cardiology, University Medical Center of the Johannes Gutenberg-University Mainz, Mainz, Germany; ^3^Center for Thrombosis and Hemostasis, University Medical Center of the Johannes Gutenberg-University Mainz, Mainz, Germany; ^4^DZHK (German Center for Cardiovascular Research), Partner Site Rhine-Main, Mainz, Germany; ^5^Department of Biomedical Statistics, University Medical Center of the Johannes Gutenberg-University Mainz, Mainz, Germany; ^6^Institute for Clinical Chemistry and Laboratory Medicine, University Medical Center of the Johannes Gutenberg-University Mainz, Mainz, Germany; ^7^Department of Psychosomatic Medicine and Psychotherapy, University Medical Center of the Johannes Gutenberg-University Mainz, Mainz, Germany; ^8^Center for Cardiology, University Medical Center of the Johannes Gutenberg-University Mainz, Mainz, Germany

## Abstract

**Purpose:**

To report the distribution of iris conicity (steepness of the iris cone), investigate associated factors, and test whether pseudophakia allows the iris to sink back.

**Methods:**

A population-based cross-sectional study was carried out. Ophthalmological examination including objective refraction, biometry, noncontact tonometry, and Scheimpflug imaging (Pentacam®, Oculus) was performed including automated measurement of iris conicity. 3708 phakic subjects, 144 subjects with bilateral and 39 subjects with unilateral pseudophakia were included. Multivariable analyses were carried out to determine independently associated systemic and ocular factors for iris conicity in phakic eyes.

**Results:**

Mean iris conicity was 8.28° ± 3.29° (right eyes). Statistical analysis revealed associations between steeper iris conicity and shallower anterior chamber depth, thicker human lens and higher corneal power in multivariable analysis, while older age was related to a flatter iris conicity. Refraction, axial length, central corneal thickness, pupil diameter, and intraocular pressure were not associated with iris conicity. Pseudophakia resulted in a 5.82° flatter iris conicity than in the fellow phakic eyes.

**Conclusions:**

Associations indicate a correlation between iris conicity with risk factors for angle-closure, namely, shallower anterior chamber depth and thicker human lens. In pseudophakic eyes, iris conicity is significantly lower, indicating that cataract surgery flattens the iris.

## 1. Introduction

Early in the history of glaucomatology, the geometry of the iris has been identified as an important factor for the understanding of aqueous humor outflow pathology, namely, for the risk of angle-closure glaucoma [1] and for the development of pigment-dispersion syndrome [2].

For almost a century, the analysis of the iris structure was restricted to examination by slit-lamp examination and gonioscopy. With the development of ultrasound biomicroscopy, quantitative examination of the anterior segment and the iris profile became possible [3, 4]. Scheimpflug imaging and anterior-segment optical coherence tomography (AS-OCT) enable the quantitative assessment of the anterior segment, and the iris geometry acquired by noncontact examination [5, 6].

The traditional mode of thinking as trained in en face gonioscopic observation of the chamber angle was transferred into the quantitative assessment of the chamber angle revealing at the same time the restrictions of such analysis by demonstrating that the chamber angle is not a geometrically well-defined entity but rather a steeply curved surface. Our study would like to open a larger view making full use of anterior segment cross-sectional imaging modalities by introducing iris conicity as a novel term into the discussion of the anatomical architecture.

The geometry of the iris is important in several anterior segment pathologies: convex iris configuration is reported in patients with primary angle-closure, is linked to age, and is inversely to anterior chamber depth [7]. On the other hand, concave iris configuration is reported in patients with pigment dispersion syndrome and is investigated with different techniques, such as AS-OCT or ultrasound biomicroscopy [8–10].

The purpose of this study is to test the potential of Scheimpflug imaging for the assessment of the iris position in the anterior chamber (iris conicity) within an epidemiological study, but not to analyze the shape of the iris (i.e., iris convexity). The relationship between Scheimpflug imaging of the iris position and anthropometric parameters, as well as parameters of the anterior segment, is analyzed. Our hypothesis is that hyperopia is linked to a steeper conicity of the entire iris, while pseudophakia allows the iris to sink back from the cornea. The rationale behind this hypothesis is that patients with acute angle-closure are more likely to by hyperopic and a shallower anterior chamber angle might be linked to a steeper iris cone. The idea behind the “pseudophakia-hypothesis” was a recent finding by the EAGLE study [11]. This study showed that clear lens extraction is able to treat angle-closure comparable or even better than standard treatment with laser iridotomy. In addition, Siak et al. [12] reported an opening of the anterior chamber angle after cataract surgery. Therefore, one can assume that the iris sinks back by cataract surgery.

## 2. Materials and Methods

The Gutenberg Health Study (GHS) is a population-based, prospective, observational cohort study conducted in the Rhine-Main region in Midwestern Germany.

This study was approved by the ethics committee (Ethics Commission of the State Chamber of Physicians of Rhineland-Palatinate). According to the tenets of the Declaration of Helsinki, written informed consent was obtained from all participants prior to entering the study. The GHS is a joint project of internal medicine, ophthalmology, clinical chemistry, psychosomatic medicine, and epidemiology at the Johannes Gutenberg-University Mainz, Germany.

### 2.1. Study Sample

This study sample was recruited from the five-year follow-up of the GHS cohort including subjects with an age of 40 to 80 years at the time of examination. For the 5-year re-examination, Scheimpflug imaging was added. We included the first third of the total study population in this analysis; this study proportion was designed to be the representative for the region of Mainz/Mainz-Bingen at baseline examination.

We included 6138 eyes of 3708 phakic subjects (48.4% women) with a mean age of 58.7 ± 10.4 years (range 40 to 80 years) having data on Scheimpflug imaging. A detailed description of the systemic and ocular characteristics of the study population is shown in Table 1. Participants who only had cataract surgery in the past were included in the pseudophakic study group.

### 2.2. Exclusion Criteria

Participants with previous ocular surgery including cataract surgery were excluded from the general distribution and association analysis. Subjects with an exclusive history of cataract surgery were included in the pseudophakia study group.

### 2.3. Examinations

For each participant, a comprehensive ophthalmological work-up was performed including anterior segment Scheimpflug imaging (Pentacam HR, Oculus, Wetzlar, Germany) under mesopic light conditions and analysis of iris position. In addition, objective refraction (Humphrey Automated Refractor/Keratometer (HARK) 599, Carl Zeiss Meditec AG, Jena, Germany), and biometry (Lenstar LS900, Haag-Streit Diagnostics, Koeniz, Switzerland) were performed. One scan was performed per eye with each of these devices always starting with the right eye. Noncontact tonometry (Nidek NT-2000, Nidek Co., Japan) was carried out also starting with the right eye. The mean of three measurements within a 3 mmHg range was obtained for each eye. Examinations were performed by experienced study nurses in accordance with standardized operation procedures. More details of the ophthalmological study design were described by Hohn et al. [13].

Age was calculated as the difference between date of birth and date of examination. Date of birth, sex, and smoking habits were surveyed in a computer-assisted personal interview. Body height and body weight measures were performed with calibrated digital scales (Seca 862, Seca, Hamburg, Germany) and a measuring stick (Seca 220, Seca, Hamburg, Germany).

### 2.4. Data and Statistical Analysis

**Figure 1:**
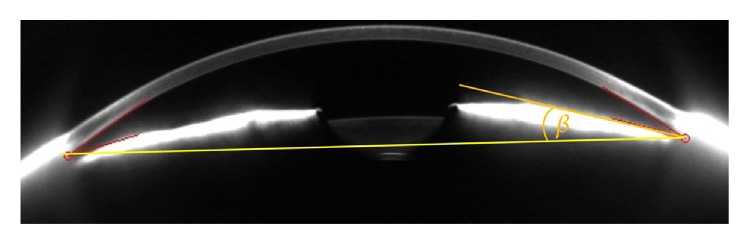
Illustration of iris conicity measurement. The iris surface is approximated by linear slopes (orange line). For illustration purposes, iris conicity is demonstrated. The opposite anterior chamber angles are connected by a plane (yellow line). Iris conicity is defined as the angle beta between these two lines. This corresponds to Pentacam “iris convexity” measure. Pentacam “iris convexity” is defined as half of the intersection angle of the linear slopes through the anterior surface of opposite iris cross-sections.

The Pentacam Scheimpflug imaging device comes with a software tool (Pentacam, v1.20r41, Oculus, Wetzlar, Germany) to measure a parameter termed “iris convexity”: This is programmed to draw a straight line across the anterior profile of the iris in a way to reach equal areas under the curve above and behind this level. The crossing angle alpha of the lines of the opposite iris profiles is divided by two to reach a figure which is meant to describe “iris convexity” (Oculus, Wetzlar, Germany, personal communication). From a geometrical point of view, this describes the conicity of the iris, that is, the mean circular slope of the iris against the connecting line between the opposed chamber angles, which is mathematically the slant angle of a truncated cone (Figure 1). This means that a low value describes a gently inclined cone, while a high value represents a steep cone. We decided to use the term conicity as it is more apt to describe the architecture of the anterior segment, while convexity by definition describes the curvature of a surface. This parameter is calculated as mean value of the Scheimpflug images.

Only Scheimpflug images with high quality were included, and all Scheimpflug measurements with a low value in the Pentacam quality score were excluded. A plausibility check was performed for all extreme values (“iris convexity” < −10° and “iris convexity” > 15°). Additionally, centration of the Scheimpflug imaging on the central cornea and opening of the eyelids were checked. Pupil size was measured simultaneously with Scheimpflug imaging. Central corneal thickness, corneal power, anterior chamber depth, lens thickness, and axial length were measured with Lenstar LS900 (Haag-Streit Diagnostics, Koeniz, Switzerland). Refraction (Humphrey Automated Refractor/Keratometer (HARK) 599, Carl Zeiss Meditec AG, Jena, Germany) was included as spherical equivalent in the analysis.

Data were processed by statistical analysis software (R version 3.3.1 [June 21, 2016]). Medians and interquartile ranges were calculated for all variables. For variables found to be nearly normally distributed, means and standard deviations were computed. Pearson's correlation coefficients were computed comparing right to left eyes with all primary and secondary variables.

Distribution of the iris conicity was evaluated using histograms. Associated factors were evaluated using linear regression models with generalized estimating equation (GEE) with consideration of the correlation structure between both eyes of the subjects. This model is applied to estimate the parameters of a generalized linear model.

We performed a three-step analysis: in the first model, sex, age, body height, body weight, and smoking status were included as independent variables to investigate associations to general anthropometric characteristics. In the second model, we include general ocular characteristics (intraocular pressure and spherical equivalent) as well. In the third model, anthropometric and biometric characteristics of the eye and intraocular pressure were included: The independent variables in this model were sex, age, height, weight, smoking, central corneal thickness, intraocular pressure, pupil diameter, corneal power of the steep meridian, anterior chamber depth, lens thickness, and axial length. Measurement of refraction was not included in this model due to collinearity. Multicollinearity was investigated by exploring pairwise Pearson's correlations.

Sensitivity analysis was carried out to determine the effect of refractive error by including only emmetropic eyes (sphere: −0.5 D ≤ ×≤0.5 D), myopic eyes (sphere < −0.5 D), and hyperopic eyes (sphere > 0.5 D). In addition, the impact of astigmatism was evaluated with including only subjects with astigmatism ≤ −1.0 D and with >−1.0 D. In addition, we included corneal power instead of refraction in the multivariable model to evaluate the influence of image projection of the cornea.

This study was performed as an explorative study to analyze distribution and associations with iris conicity. All *p* values should be regarded as a continuous parameter which reflect the level of statistical evidence and are therefore reported exactly.

## 3. Results

**Figure 2:**
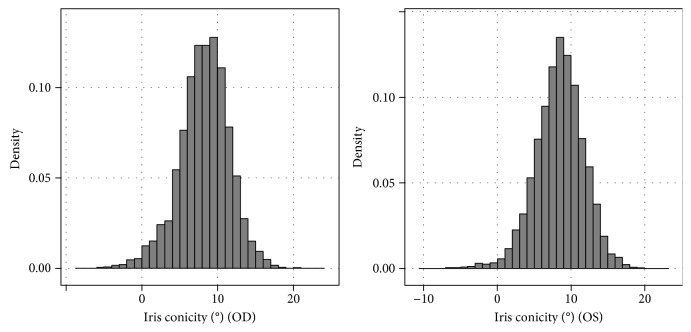
Distribution of iris conicity (“iris convexity,” Pentacam) in the German population: the Gutenberg Health Study. (a) Right eyes. (b) Left eyes. Density is displayed as the proportion to the total study population (right eyes: 3060; left eyes: 3078).

Mean iris conicity was 8.28° ± 3.29° (right eyes) and 8.51° ± 3.27° with a range from −7° to 22° (Figure 2). A negative iris conicity indicates a positioning of the iris surface backwards (towards the vitreous cavity).

Comparing right to left eyes, iris conicity was highly correlated between both sides (*p* < 0.001), showing a correlation coefficient of 0.83.

Associated factors with the iris conicity were examined by a generalized estimating equation model and including only phakic eyes. The first model using sex, age, height, weight, and smoking status as independent variables showed sex (*p* = 0.004) and age (*p* < 0.001) as associated factors. The measurement of the conicity revealed a 0.35° (95% confidence interval (CI): −0.59 to −0.11; *p* = 0.004) lower inclination in women compared to men. Older subjects had a slightly flatter inclination. Each decade of age was associated with a 0.18° smaller iris conicity (per 10 years: beta = −0.18; 95% CI: −0.27 to −0.09; *p* < 0.001).

The second analysis model including anthropometric characteristics and general ocular characteristics, namely, intraocular pressure and refraction (spherical equivalent) showed the same associated factors as in model number 1 (Table 2), neither refraction nor intraocular pressure was associated.

The third analysis also included biometrics of the eye. It revealed that a shallower anterior chamber, higher corneal power, a thicker human lens, and younger age were associated with a steeper inclination of the iris (conicity). There was no common association with body height, body weight, smoking status, central corneal thickness, axial length, intraocular pressure, and pupil diameter in this multivariable model (Table 3). Pairwise correlation coefficients were low, indicating that there is no clear evidence for multicollinearity.

Sensitivity analysis revealed that association of iris conicity with age and lens thickness is independent of refractive status: comparable associations were found in the myopic, emmetropic, hyperopic subgroup, as in the subgroup with high astigmatism (Table 4). Interestingly, axial length was only associated with iris conicity in emmetropic and hyperopic eyes, but not in myopic eyes. In contrast, anterior chamber depth was only associated with iris conicity in the myopic subgroup, but not in the other subgroups.

Anterior chamber angle was inversely associated with iris conicity in phakic eyes (Pearson's correlation coefficient: *r* = −0.46, *p* < 0.001).

Mean iris conicity was 0.74 ± 2.52° in right pseudophakic eyes and 0.77 ± 2.61° in left pseudophakic eyes. When comparing right phakic (*n* = 2914) to right pseudophakic eyes (*n* = 135), iris conicity was 7.54° lower in the pseudophakic eyes (*p* < 0.001). A similar finding was detected in left eyes: iris conicity was 7.74° lower in pseudophakic left eyes (*n* = 144) compared to phakic left eyes (*n* = 2936; *p* < 0.001).

**Figure 3:**
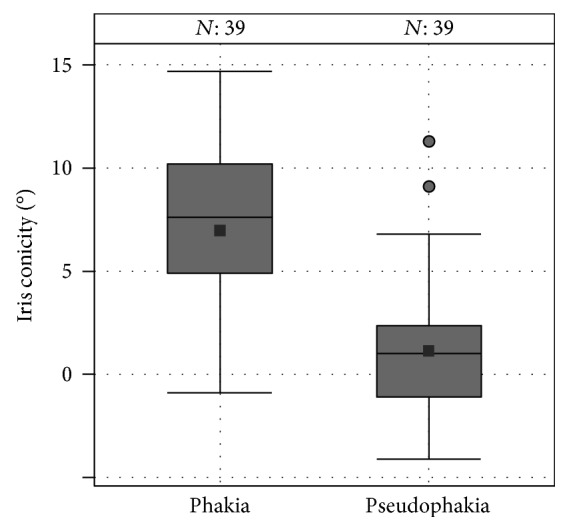
Box plots of iris conicity of intraindividual comparison of phakic and pseudophakic eyes in the GHS study population (*N* is the number of subjects with one eye having pseudophakia and the fellow eye being phakic in our study population).

**Figure 4:**
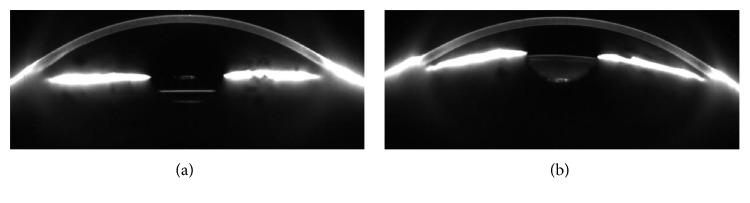
Example of Scheimpflug images of an intraindividual comparison of a pseudophakic right eye (a) and a phakic left eye (b) in the GHS study population. Please observe the different light reflexes by the artificial intraocular lens and the crystalline lens. The iris is flat in the pseudophakic eye (a), while it is positioned towards the cornea in the phakic eye (b). This is described by iris conicity: iris conicity was −1° in the pseudophakic eye and 11° in the phakic eye.

When analyzing subjects with one phakic eye and the fellow eye being pseudophakic (*n* = 39), iris conicity was 5.82° smaller in the pseudophakic eyes (95% confidence interval: −7.66°; −3.98°; paired *t* test: *p* < 0.001) (Figure 3()). An example is given in Figure 4().

## 4. Discussion

To the best of our knowledge, this is the first study to quantitatively analyze iris conicity in a population-based setting using Scheimpflug imaging. We found a mean iris conicity of 8.28° ± 3.29° for right eyes and 8.51° ± 3.27° for left eyes.

When analyzing anthropometric and ocular parameters (refraction, intraocular pressure), the model showed female gender and higher age as independent associated factors with a flatter iris conicity in the multivariable model, showing that both are related to iris conicity. To our surprise, refraction was not found to be associated with iris conicity. Corneal curvature was associated with iris conicity: a 10-diopter higher corneal power was associated with a 0.9° steeper iris inclination which may, however, not be of clinical relevance.

While several studies investigated anterior chamber angle width using Scheimpflug imaging, AS-OCT, or ultrasound biomicroscopy [14–19], little is known about the iris position in the anterior segment. The anterior chamber angle is formed by the posterior corneal surface and the peripheral anterior iris surface and therefore a close association between a steeper forward inclination of the iris profile and a smaller anterior chamber angle may appear likely. In agreement with this consideration, we found an inverse correlation between anterior chamber angle width and iris conicity.

Bearing this close correlation in mind, findings of our third analytical model investigating biometric parameters of the eye seem plausible. A more marked forward slope (steeper iris conicity) was independently associated with a shallower anterior chamber, higher corneal power, and a thicker crystalline lens. Similar findings for a smaller anterior chamber angle were previously reported by other groups [20, 21]. These results are similar to observations in patients with angle-closure glaucoma who tend to have a shallower anterior chamber and a thicker crystalline lens [22]. While a shallower anterior chamber depth was related to a steeper iris conicity in the main analysis, stratification on refractive error did yield that this is primarily visible in myopic eyes but not in emmetropic and hyperopic eyes. A decisive geometrical parameter may be the distance between the iris pigment epithelium and the anterior capsule of the crystalline lens. This however may not be approached by current noncontact imaging methods. We hence cannot state whether the observed iris conicity is determined by the flow of the aqueous humor between the anterior surface of the lens and the posterior surface of the iris or rather as a primary anatomical parameter by the intrinsic configuration of iris tissue.

Interestingly, axial length was not associated with iris conicity in the main analysis, while several studies reported an association between shorter axial length and a smaller anterior chamber angle and angle-closure glaucoma [15, 18, 23]. Nevertheless, subgroup analysis revealed an association in emmetropic and hyperopic eyes but not in myopic eyes, indicating that there might be a relationship in the physiological range of axial length. One may speculate that myopia is mainly linked to a relative elongation of the posterior segment of the eye, and therefore, a direct association may remain elusive. Also, the position of the iris root may be a determinant for the absolute conicity of the iris in so far as the accommodative state of the ciliary muscle may exert a subtle deformation. Moreover, it remains speculative whether the actual thickness of the crystalline lens is larger in a hyperopic eye compared to a myopic eye of the same absolute refractive error.

Similarly, iris conicity was not associated with central corneal thickness which is known to be linked with anterior chamber angle aperture [19, 24]. These findings indicate that iris conicity follows other rules than the mere chamber angle. While the anterior chamber angle is measured peripherally, the iris conicity indicates a spatial position of the iris in the anterior segment. However, both parameters are a geometrical reduction of complex microanatomical structure which do not lend themselves easily to a description by two straight crossing lines.

Iris configuration rather than iris position was investigated in several other studies. Iris concavity was reported to be present in pigment dispersion syndrome and pigmentary glaucoma [8]. In those eyes, peripheral sagging of the iris tissue is so marked that it leads to chafing of the pigment epithelium on the reverse side against the anterior lens capsule and zonular fibres [9]. Moderate myopia is associated with such a concave iris configuration [9, 10]. Iris contour itself is known to be changed by several physiological mechanisms, such as blinking [3], accommodation [25–28], and exercise [29, 30]. In addition, dynamic analysis of iris configuration shows changes in dark and bright light conditions [31]. Whether these factors do also influence iris position in the anterior segment is not fully understood.

There are methodological limitations to the study. First, we were not able to adjust for the potential influence of the pupil diameter on iris conicity under different lighting conditions. Imaging of the anterior segment was performed under mesopic lighting conditions without dilation of the pupil. Nevertheless, Scheimpflug imaging uses bright light source. Furthermore, we did not assess objective refraction under cycloplegic conditions; therefore, findings with respect to hyperopia might be underestimated. Also, accommodation might have influenced our results on lens thickness. However, our study cohort had an age range from 40 to 80 years. In the older age groups, the geometrical effect of accommodation on the lens configuration is limited. Also, we reported a cross-sectional analysis of associations with iris conicity and did not refer to actual changes of the iris geometry over time. Therefore age-related changes do not refer to individual changes over time. A critical look should be taken at the measurement method. We used the built-in measurement tool and performed a plausibility check for inappropriate values. Furthermore, the iris structure itself is approximated by a linear slope and intersection of opposite slopes are used to calculate the iris conicity. This measurement does not incorporate the iris profile (convex or concave configuration, plateau iris) but approximates the average iris position in the anterior segment. While this algorithm is less prone to errors than the identification of the anterior chamber angle, its value in glaucomatology is not yet established. In addition, we did not analyze a real object, namely, the iris, but its image projected by the cornea. Sensitivity analysis with incorporation of corneal power did not alter our findings. As most of our study participants were Caucasians, our conclusions should be regarded as valid for this ethnicity only.

The distribution of ocular parameters in our study population should be considered when interpreting our findings. Mean central corneal thickness (right eyes: 549 *μ*m) was higher in the German population than in other studies [32, 33], as previously discussed [34].

Anterior chamber depth was also slightly shallower when comparing to literature. Our study showed a mean anterior chamber depth of 2.70 mm (right eyes), while Pan et al. [35] reported mean measurements of 3.15 mm for anterior chamber depth in Indian ethnicity and Lim et al. [36] 3.10 mm in Malay ethnicities. Axial length was comparable to other studies analyzing urban populations [35, 36], while in rural China and central India, axial length is shorter [37, 38]. With regard to lens thickness, our distribution was similar to literature when taking age distribution into account. The Central India Eye and Medical Study reported a lens thickness of 3.95 mm analyzing participants being a decade younger [39]. In a Burmese population for instance, lens thickness is slightly thicker (mean lens thickness 4.52 mm) than in our study [40]. Measurements of intraocular pressure are lower in our study compared to literature, as described for the baseline examination by Hoehn et al. [41] and being also true in the follow-up examination. Overall, distributions of ocular parameters in our study population were comparable to urban population, except for intraocular pressure.

In summary, we report the distribution of iris conicity using Scheimpflug imaging in a population-based study. A steeper conicity was independently associated with a shallow anterior chamber, and a thicker crystalline lens, while older persons had a flatter iris conicity. In pseudophakic eyes, iris conicity approaches 0°, showing that cataract surgery flattens the iris position.

## Figures and Tables

**Table 1:** Characteristics of the study sample of the Gutenberg Health Study. Means (standard deviations) and proportions in percentage (*n*/*N*) for dichotomous variables in the total cohort for males and females. CCT: central corneal thickness; IOP: intraocular pressure; OD: right eyes; OS: left eyes.

Variable	All (3708)	Men (1913)	Women (1795)
Sex (women)	48.4% (1795/3708)	0% (0/1913)	100.0% (1795/1795)
Age (y)	58.7 (10.4)	59.2 (10.4)	58.1 (10.3)
Smoking (yes)	16.4% (605/3680)	16.7% (318/1899)	16.1% (287/1781)
Body mass index (kg/m^2^)	27.5 (4.8)	27.9 (4.2)	27.0 (5.4)
Height (m)	1.70 (0.09)	1.77 (0.07)	1.64 (0.07)
Weight (kg)	80.0 (16.2)	87.3 (14.2)	72.1 (14.5)
*Eyes*			
Spherical equivalent (dpt) (OD)	−0.41 (2.51)	−0.44 (2.52)	−0.38 (2.49)
Spherical equivalent (dpt) (OS)	−0.42 (2.53)	−0.46 (2.55)	−0.37 (2.52)
CCT (*μ*m) (OD)	549 (35)	553 (35)	546 (35)
CCT (*μ*m) (OS)	550 (35)	552 (35)	547 (35)
IOP (mmHg) (OD)	14.88 (2.93)	14.97 (3.00)	14.78 (2.85)
IOP (mmHg) (OS)	14.99 (2.95)	15.17 (2.98)	14.80 (2.91)
Axial length (mm) (OD)	23.7 (1.2)	24.0 (1.2)	23.4 (1.2)
Axial length (mm) (OS)	23.7 (1.3)	24.0 (1.3)	23.4 (1.2)
Lens thickness (mm) (OD)	4.37 (0.36)	4.38 (0.37)	4.36 (0.35)
Lens thickness (mm) (OS)	4.42 (0.35)	4.44 (0.36)	4.41 (0.34)
Corneal power (dpt) (OD)	44.0 (1.6)	43.6 (1.6)	44.4 (1.5)
Corneal power (dpt) (OS)	44.0 (1.6)	43.6 (1.6)	44.4 (1.5)
Anterior chamber depth (mm) (OD)	2.70 (0.36)	2.75 (0.37)	2.64 (0.34)
Anterior chamber depth (mm) (OS)	2.69 (0.36)	2.74 (0.36)	2.63 (0.35)
Mean pupil diameter (mm) (OD)	2.69 (0.45)	2.63 (0.44)	2.75 (0.46)
Mean pupil diameter (mm) (OS)	2.67 (0.44)	2.61 (0.42)	2.73 (0.45)
*Iris conicity*			
Iris conicity mean (°) (OD)	8.28 (3.29)	8.45 (3.26)	8.09 (3.31)
Iris conicity mean (°) (OS)	8.51 (3.27)	8.65 (3.22)	8.36 (3.32)

**Table 2:** Associations of iris conicity and anthropometric characteristics and intraocular pressure (IOP) and refraction in the Gutenberg Health Study using a generalized estimating equation model to incorporate correlations between right and left eyes. CI: confidence interval.

Iris conicity (°)	Beta estimate	Lower 95% CI	Upper 95% CI	*p* value
Sex (female)	−0.36	−0.61	−0.11	**0.005**
Age (y)	−0.02	−0.02	−0.01	**0.00**2
Height (m)	−0.59	−2.00	0.83	0.42
Weight (kg)	0.00	−0.01	0.01	0.95
Smoking	0.25	0.01	0.49	**0.045**
Intraocular pressure (mmHg)	−0.02	−0.05	0.01	0.18
Spherical equivalent (dpt)	0.00	−0.04	0.04	0.95

**Table 3:** Associations of iris conicity and anthropometric and ocular characteristics including ocular geometric parameters in the Gutenberg Health Study. We used a generalized estimating equation model to consider correlations between right and left eyes in our statistical model. CI: confidence interval.

Iris conicity (°)	Beta estimate	Lower 95% CI	Upper 95% CI	*p* value
Sex (female)	−0.32	−0.56	−0.07	**0.011**
Age (y)	−0.07	−0.08	−0.06	**<0.001**
Height (m)	0.99	−0.40	2.38	0.16
Weight (kg)	0.00	0.00	0.01	0.51
Smoking	0.03	−0.20	0.26	0.79
Intraocular pressure (mmH)	−0.03	−0.07	0.00	0.052
Corneal power (dpt)	0.09	0.03	0.16	**0.003**
Anterior chamber depth (mm)	−0.71	−1.05	−0.37	**<0.001**
Central corneal thickness (*μ*m)	0.00	0.00	0.00	0.53
Lens thickness (mm)	2.77	2.43	3.10	**<0.001**
Axial length (mm)	−0.05	−0.15	0.04	0.26
Mean pupil diameter (*μ*m)	−0.10	−0.31	0.11	0.34

**Table:** (a) Analysis of myopic subjects (*N* = 1688 eyes)

Iris conicity (°) ~	Beta estimate	Lower 95% CI	Upper 95% CI	*p* value
Sex	−0.45	−0.88	−0.02	**0.04**
Age (y)	−0.08	−0.10	−0.06	**<0.0001**
Height (m)	1.75	−0.63	4.14	0.15
Weight (kg)	0.00	−0.01	0.01	0.53
Smoking	0.23	−0.21	0.67	0.31
IOP (mmHg)	−0.02	−0.08	0.04	0.48
Corneal power (dpt)	0.04	−0.07	0.15	0.46
Anterior chamber depth (mm)	−0.77	−1.36	−0.17	**0.01**
CCT (*μ*m)	−0.00	−0.01	0.00	0.40
Lens thickness (mm)	2.57	2.00	3.14	**<0.0001**
Axial length (mm)	−0.04	−0.20	0.13	0.67
Mean pupil diameter (mm)	−0.22	−0.59	0.15	0.25

**Table:** (b) Analysis of emmetropic subjects (*N* = 1613 eyes)

Iris conicity (°) ~	Beta estimate	Lower 95% CI	Upper 95% CI	*p* value
Sex	−0.15	−0.60	0.30	0.52
Age (y)	−0.06	−0.08	−0.04	**<0.0001**
Height (m)	1.77	−0.86	4.40	0.19
Weight (kg)	0.01	−0.01	0.02	0.30
Smoking	−0.31	−0.69	0.06	0.10
IOP (mmHg)	−0.02	−0.08	0.04	0.54
Corneal power (dpt)	−0.09	−0.25	0.08	0.31
Anterior chamber depth (mm)	−0.31	−0.97	0.36	0.37
CCT (*μ*m)	0.00	−0.00	0.01	0.59
Lens thickness (mm)	3.02	2.37	3.66	**<0.0001**
Axial length (mm)	−0.56	−0.95	−0.16	**0.006**
Mean pupil diameter (mm)	−0.08	−0.46	0.30	0.69

**Table:** (c) Analysis of hyperopic subjects (*N* = 1980 eyes)

Iris conicity (°) ~	Beta estimate	Lower 95% CI	Upper 95% CI	*p* value
Sex	−0.51	−0.90	−0.11	**0.01**
Age (y)	−0.06	−0.07	−0.04	**<0.0001**
Height (m)	0.10	−2.17	2.36	0.93
Weight (kg)	−0.00	−0.01	0.01	0.80
Smoking	0.28	−0.11	0.67	0.15
IOP (mmHg)	−0.06	−0.11	−0.01	**0.02**
Corneal power (dpt)	−0.05	−0.17	0.08	0.49
Anterior chamber depth (mm)	−0.37	−0.96	0.23	0.23
CCT (*μ*m)	−0.00	−0.01	0.00	0.47
Lens thickness (mm)	2.70	2.18	3.22	**<0.0001**
Axial length (mm)	−0.55	−0.80	−0.30	**<0.0001**
Mean pupil diameter (mm)	−0.09	−0.43	0.25	0.59

**Table:** (d) Analysis of subjects with high astigmatism (*N* = 659 eyes)

Iris conicity (°) ~	Beta estimate	Lower 95% CI	Upper 95% CI	*p* value
Sex	0.13	−0.61	0.85	0.74
Age (y)	−0.10	−0.13	−0.07	**<0.0001**
Height (m)	1.76	−2.31	5.84	0.40
Weight (kg)	0.00	−0.01	0.02	0.90
Smoking	0.18	−0.48	0.83	0.60
IOP (mmHg)	0.04	−0.05	0.13	0.42
Corneal power (dpt)	0.09	−0.06	0.23	0.25
Anterior chamber depth (mm)	−0.89	−1.80	0.02	0.056
CCT (*μ*m)	−0.01	−0.02	−0.00	**0.03**
Lens thickness (mm)	2.97	2.01	3.94	**<0.0001**
Axial length (mm)	0.08	−0.13	0.30	0.45
Mean pupil diameter (mm)	−0.07	−0.65	0.51	0.81

## References

[1] Aung T., Nolan W. P., Machin D. (2005). Anterior chamber depth and the risk of primary angle closure in 2 East Asian populations. *Archives of Ophthalmology*.

[2] Campbell D. G., Schertzer R. M. (1995). Pathophysiology of pigment dispersion syndrome and pigmentary glaucoma. *Current Opinion in Ophthalmology*.

[3] Liebmann J. M., Tello C., Chew S. J., Cohen H., Ritch R. (1995). Prevention of blinking alters iris configuration in pigment dispersion syndrome and in normal eyes. *Ophthalmology*.

[4] Pavlin C. J., Harasiewicz K., Foster F. S. (1995). An ultrasound biomicroscopic dark-room provocative test. *Ophthalmic Surgery*.

[5] Liu L., Ong E. L., Crowston J. (2011). The concave iris in pigment dispersion syndrome. *Ophthalmology*.

[6] Schuster A. K., Fischer J. E., Vossmerbaeumer U. (2017). Curvature of iris profile in spectral domain optical coherence tomography and dependency to refraction, age and pupil size - the MIPH Eye&Health Study. *Acta Ophthalmologica*.

[7] Nonaka A., Iwawaki T., Kikuchi M., Fujihara M., Nishida A., Kurimoto Y. (2007). Quantitative evaluation of iris convexity in primary angle closure. *American Journal of Ophthalmology*.

[8] Kanadani F. N., Dorairaj S., Langlieb A. M. (2006). Ultrasound biomicroscopy in asymmetric pigment dispersion syndrome and pigmentary glaucoma. *Archives of Ophthalmology*.

[9] Aptel F., Beccat S., Fortoul V., Denis P. (2011). Biometric analysis of pigment dispersion syndrome using anterior segment optical coherence tomography. *Ophthalmology*.

[10] Asrani S., Sarunic M., Santiago C., Izatt J. (2008). Detailed visualization of the anterior segment using fourier-domain optical coherence tomography. *Archives of Ophthalmology*.

[11] Azuara-Blanco A., Burr J., Ramsay C. (2016). Effectiveness of early lens extraction for the treatment of primary angle-closure glaucoma (EAGLE): a randomised controlled trial. *Lancet*.

[12] Siak J., Quek D., Nongpiur M. E. (2016). Anterior chamber angle and intraocular pressure changes after phacoemulsification: a comparison between eyes with closed-angle and open-angle glaucoma. *Journal of Glaucoma*.

[13] Hohn R., Kottler U., Peto T. (2015). The ophthalmic branch of the Gutenberg health study: study design, cohort profile and self-reported diseases. *PLoS One*.

[14] Barkana Y., Dekel I., Goldich Y., Morad Y., Avni I., Zadok D. (2012). Angle closure in Caucasians—a pilot, general ophthalmology clinic-based study. *Journal of Glaucoma*.

[15] Kim Y. Y., Lee J. H., Ahn M. D., Kim C. Y., Namil Study Group, Korean Glaucoma Society (2012). Angle closure in the Namil study in Central South Korea. *Archives of Ophthalmology*.

[16] Senthil S., Garudadri C., Khanna R. C., Sannapaneni K. (2010). Angle closure in the Andhra Pradesh eye disease study. *Ophthalmology*.

[17] van Romunde S. H., Thepass G., Lemij H. G. (2013). Is hyperopia an important risk factor for PACG in the Dutch population?-a case control study. *Journal of Ophthalmology*.

[18] Vijaya L., George R., Arvind H. (2006). Prevalence of angle-closure disease in a rural southern Indian population. *Archives of Ophthalmology*.

[19] Schuster A. K., Pfeiffer N., Nickels S. (2016). Distribution of anterior chamber angle width and correlation with age, refraction, and anterior chamber depth-the Gutenberg health study. *Investigative Ophthalmology & Visual Science*.

[20] Hashemi H., Khabazkhoob M., Mohazzab-Torabi S. (2016). Anterior chamber angle and anterior chamber volume in a 40- to 64-year-old population. *Eye & Contact Lens*.

[21] Fernandez-Vigo J. I., Fernandez-Vigo J. A., Macarro-Merino A., Fernández-Pérez C., Martínez-de-la-Casa J. M., García-Feijoó J. (2016). Determinants of anterior chamber depth in a large Caucasian population and agreement between intra-ocular lens Master and Pentacam measurements of this variable. *Acta Ophthalmologica*.

[22] Yazdani S., Akbarian S., Pakravan M., Doozandeh A., Afrouzifar M. (2015). Biometric parameters in different stages of primary angle closure using low-coherence interferometry. *Optometry and Vision Science*.

[23] Lowe R. F. (1970). Aetiology of the anatomical basis for primary angle-closure glaucoma. Biometrical comparisons between normal eyes and eyes with primary angle-closure glaucoma. *The British Journal of Ophthalmology*.

[24] Xu L., Cao W. F., Wang Y. X., Chen C. X., Jonas J. B. (2008). Anterior chamber depth and chamber angle and their associations with ocular and general parameters: the Beijing eye study. *American Journal of Ophthalmology*.

[25] Adam R. S., Pavlin C. J., Ulanski L. J. (2004). Ultrasound biomicroscopic analysis of iris profile changes with accommodation in pigmentary glaucoma and relationship to age. *American Journal of Ophthalmology*.

[26] Dorairaj S., Oliveira C., Fose A. K. (2008). Accommodation-induced changes in iris curvature. *Experimental Eye Research*.

[27] Pavlin C. J., Harasiewicz K., Foster F. S. (1994). Posterior iris bowing in pigmentary dispersion syndrome caused by accommodation. *American Journal of Ophthalmology*.

[28] Balidis M. O., Bunce C., Sandy C. J., Wormald R. P., Miller M. H. (2002). Iris configuration in accommodation in pigment dispersion syndrome. *Eye*.

[29] Jensen P. K., Nissen O., Kessing S. V. (1995). Exercise and reversed pupillary block in pigmentary glaucoma. *American Journal of Ophthalmology*.

[30] Haargaard B., Jensen P. K., Kessing S. V., Nissen O. I. (2001). Exercise and iris concavity in healthy eyes. *Acta Ophthalmologica Scandinavica*.

[31] Cheung C. Y., Liu S., Weinreb R. N. (2010). Dynamic analysis of iris configuration with anterior segment optical coherence tomography. *Investigative Ophthalmology & Visual Science*.

[32] Tomidokoro A., Araie M., Iwase A. (2007). Corneal thickness and relating factors in a population-based study in Japan: the Tajimi study. *American Journal of Ophthalmology*.

[33] Eysteinsson T., Jonasson F., Sasaki H. (2002). Central corneal thickness, radius of the corneal curvature and intraocular pressure in normal subjects using non-contact techniques: Reykjavik eye study. *Acta Ophthalmologica Scandinavica*.

[34] Hoffmann E. M., Lamparter J., Mirshahi A. (2013). Distribution of central corneal thickness and its association with ocular parameters in a large central European cohort: the Gutenberg health study. *PLoS One*.

[35] Pan C. W., Wong T. Y., Chang L. (2011). Ocular biometry in an urban Indian population: the Singapore Indian eye study (SINDI). *Investigative Ophthalmology & Visual Science*.

[36] Lim L. S., Saw S. M., Jeganathan V. S. (2010). Distribution and determinants of ocular biometric parameters in an Asian population: the Singapore Malay eye study. *Investigative Ophthalmology & Visual Science*.

[37] Fu T., Song Y. W., Chen Z. Q. (2015). Ocular biometry in the adult population in rural central China: a population-based, cross-sectional study. *International Journal of Ophthalmology*.

[38] Jonas J. B., Iribarren R., Nangia V. (2015). Lens position and age: the Central India eye and medical study. *Investigative Ophthalmology & Visual Science*.

[39] Jonas J. B., Nangia V., Gupta R., Sinha A., Bhate K. (2012). Lens thickness and associated factors. *Clinical and Experimental Ophthalmology*.

[40] Warrier S., Wu H. M., Newland H. S. (2008). Ocular biometry and determinants of refractive error in rural Myanmar: the Meiktila eye study. *The British Journal of Ophthalmology*.

[41] Hoehn R., Mirshahi A., Hoffmann E. M. (2013). Distribution of intraocular pressure and its association with ocular features and cardiovascular risk factors: the Gutenberg health study. *Ophthalmology*.

